# A Nationally Representative Survey of COVID-19 in Pakistan, 2021–2022

**DOI:** 10.3201/eid2813.220728

**Published:** 2022-12

**Authors:** Sarah Aheron, Kerton R. Victory, Amnah Imtiaz, Ian Fellows, Sara I. Gilani, Bilal Gilani, Christie Reed, Avi J. Hakim

**Affiliations:** US Centers for Disease Control & Prevention, Pretoria, South Africa (S. Aheron);; US Centers for Disease Control & Prevention, Atlanta, Georgia, USA (K.R. Victory, A.J. Hakim);; Gallup Pakistan, Islamabad, Pakistan (A. Imtiaz, S.I. Gilani, B. Gilani);; Fellows Statistics, San Diego, CA, USA (I. Fellows);; US Centers for Disease Control & Prevention, Islamabad, Pakistan (C. Reed)

**Keywords:** COVID-19, Pakistan, survey, coronavirus disease, SARS-CoV-2, severe acute respiratory syndrome coronavirus 2, viruses, respiratory infections, zoonoses, vaccine-preventable diseases

## Abstract

We conducted 4,863 mobile phone and 1,715 face-to-face interviews of adults >18 years residing in Pakistan during June 2021–January 2022 that focused on opinions and practices related to COVID-19. Of those surveyed, 26.3% thought COVID-19 was inevitable, and 16.8% had tested for COVID-19. Survey participants who considered COVID-19 an inevitability shared such traits as urban residency, concerns about COVID-19, and belief that the virus is a serious medical threat. Survey respondents who had undergone COVID-19 testing shared similarities regarding employment status, education, mental health screening, and the consideration of COVID-19 as an inevitable disease. From this survey, we modeled suspected and confirmed COVID-19 cases and found nearly 3 times as many suspected and confirmed COVID-19 cases than had been reported. Our research also suggested undertesting for COVID-19 even in the presence of COVID-19 symptoms. Further research might help uncover the reasons behind undertesting and underreporting of COVID-19 in Pakistan.

The novel coronavirus SARS-CoV-2 was characterized as a pandemic by the World Health Organization on March 11, 2020 (*1*), after its discovery in Wuhan, China, in December 2019. The first case of COVID-19 in Pakistan was reported on February 26, 2020, with the government declaring an outbreak the same day (*2*–*5*). As of December 31, 2021, there were >1,290,000 confirmed COVID-19 cases and 28,909 COVID-19–related deaths in Pakistan (*6*).

Given the relatively young median age in Pakistan of 23 years, fewer cases of severe disease have been reported in Pakistan than in other countries, which is consistent with previously observed findings of decreased disease severity among younger persons (*7*). Pakistan is especially vulnerable to COVID-19 spread because of the country’s high population density and average household size of 6.4 persons (*8*). Sixty-two percent of residents live in rural areas with inadequate or inaccessible healthcare facilities, and many others are reluctant to access health services (*9*,*10*). Although Pakistan has infection control and prevention guidelines, including those specific to COVID-19, these guidelines were not uniformly implemented in public healthcare settings before or during COVID-19, which might have perpetuated public distrust of the healthcare system and reluctance of residents to use health services (*11*–*14*).

Administration of COVID-19 vaccines in Pakistan began in February 2021, and by December 31, 2021, a total of 31.3% of the population had completed a fully primary vaccination series, and another 11.7% were partially vaccinated. However, only those >12 years of age were eligible for vaccination (*15*). Although vaccination is a critical prevention measure, nonpharmaceutical interventions to prevent the spread of COVID-19 also are critical to ensure that the healthcare system is not overwhelmed during surges. Challenges to Pakistan’s vaccine program include scarcity of pediatric doses, introduction of booster doses, and lower efficacy of 2-dose vaccine regimens (*15*,*16*).

Individual prevention behaviors, such as physical distancing and mask wearing, can lead to decreases in the sickness and death rates related to COVID-19 (*17*,*18*). It is therefore important to understand the willingness of residents to engage in these behaviors to maximize the safety of the population. Previous studies have shown that personal perceived risk regarding COVID-19 during the pandemic varies based on such factors as demographics, physical health, anxiety about COVID-19, and knowing someone who had contracted COVID-19 (*19*,*20*). Perceived risk is an important factor for engagement in prevention behaviors (*21*). Understanding perceived risk across different groups—and how perceived risk translates to behavior—can inform policy and interventions.

Two years into the pandemic, mitigation policies and the social and emotional toll of the pandemic have left many populations weary (*22*). Because COVID-19 will remain a threat for the foreseeable future, it is important to understand its effect on society and the willingness of persons to continue engaging in prevention measures. Vaccination rates are stagnating in many countries, and immunity (natural and vaccine-derived) wanes over time. Waning immunity could leave persons vulnerable to future infection. Pandemic fatigue may also affect willingness to get tested for COVID-19, especially for those who have already been infected or vaccinated or who have seen others get infected and experience only mild symptoms. Testing remains important for detection of COVID-19 cases to facilitate isolation of infected persons and for surveillance purposes (*23*). Delays in testing can result in continued transmission; it is important therefore to understand factors that influence a person’s decision to test (*24*).

As of December 31, 2021, Pakistan ranked 102nd out of 132 countries for administered COVID-19 tests per million persons, suggesting that the number of cases reported and, in turn, the number of COVID-19–associated deaths are underestimated (*6*,*25*). We conducted a survey in Pakistan to gather nationally and provincially representative data about the knowledge and attitudes of residents regarding COVID-19 and how they are responding to the pandemic. In addition, we estimated the number of COVID-19 suspected and confirmed cases. We hope that our data will help to inform evidence-based policies and programs in Pakistan.

## Materials and Methods

### Study Design and Setting

We conducted a national cross-sectional, 2-stage, cluster survey in all 4 provinces of Pakistan (Punjab, Sindh, Kyber Pakhtunkhwa, and Balochistan), as well as other territories (Gilgit Baltistan, Azad Jamu, and Kashmir), using mobile phone and face-to-face interviews. Mobile phone interviews were conducted from June 29, 2021, through August 16, 2021. Face-to-face interviews were conducted from December 8, 2021, through January 11, 2022. Funding delays caused the gap of approximately 4 months between the end of the mobile phone survey and the beginning of the face-to-face survey. 

We determined eligibility criteria based on such chief factors as age ≥18 years, ability to speak Urdu, Pashto, or Sindhi, and willingness to provide verbal consent. The duration of interviews was ≈15 minutes for mobile phone interviews and ≈25 minutes for face-to-face interviews. We conducted the mobile phone interviews with the intent of mitigating the spread of SARS-CoV-2 throughout the country. Recognizing that mobile phone ownership is not universal in Pakistan, we carried out face-to-face interviews to supplement those conducted by mobile phone and reach underrepresented populations, such as women, those living in rural areas without mobile phone service, and persons at lower socio-economic status and thus less likely to own mobile phones.

We selected mobile phone interviewees using random digit dialing. We randomly selected phone numbers from a national repository of registered mobile phone numbers based on the proportion of market share held by mobile phone providers. If no answer, we made 3 callback attempts. Estimating a response rate of 5%, we selected 120,000 phone numbers to reach the target sample size of 4,980. Once we reached the target sample size, we stopped conducting interviews. We selected participants for the face-to-face survey using a 2-stage, stratified, cluster sampling design. In the first stage, we selected 132 primary sampling units (PSUs) from 2 strata using the 2017 census, with probability proportionate to size (*8*). The PSUs were urban census blocks and villages. In the second stage, we divided the PSUs into 4 parts with equal numbers of households. We conducted interviews using a modified Kish Grid approach to select 1 of the 4 quadrants from which households were sampled (*26*). The interviewer went to the center of the segment and randomly selected a household and then went to every fifth household using a right-hand rule. If there was >1 eligible respondent in a household, we randomly selected 1 using the Kish Grid method. We estimated the sample size for face-to-face interviews as 1,320, a number that we determined would supplement the mobile phone interviews and be large enough to be representative in terms of sex, province, age, language, education, and occupation at national and subnational levels.

### Study Instrument

We developed a questionnaire comprised of 9 modules: demographics, COVID-19 history, knowledge, attitudes, behavior, mental health, violence, the effect of COVID-19, and COVID-19 sources of information. We used the Patient Health Questionnaire 4 scale to categorize mental health status into normal (score of 0–2), mild (3–5), moderate (6–8), and severe (9–12) (*27*). A score of higher than normal indicates anxiety and depression.

### Data and Analysis

We conducted descriptive analysis to calculate the frequency and percentage of demographic information, behaviors, and perceptions. This primary analysis focused on 2 correlates: 1) belief that it is impossible to avoid contracting COVID-19 (referred to as “COVID-19 is inevitable”); and 2) history of testing for COVID-19. We also estimated the number of suspected and confirmed cases. We defined confirmed cases as someone who had a laboratory-confirmed positive COVID-19 test, and we defined suspected cases as someone who was not tested for COVID-19 but experienced COVID-19 symptoms, including new loss of taste or smell or any 3 of these symptoms: fever, cough, headache, general weakness or fatigue, sore muscles, sore throat, loss of appetite, diarrhea, and difficulty breathing.

We constructed survey weights by iteratively poststratifying the sample on educational attainment, occupation, province, rural/urban, and sex. We obtained population proportions for the poststratifications from census data (*8*). When calculating population proportions from the rate at which an event occurs in the households of the respondents, we further weighted inversely by household size, because members of large households would be overrepresented in the sample otherwise.

We conducted logistic regression to assess correlates of believing that contracting COVID-19 was inevitable and history of ever testing for COVID-19. We included variables with an association of p<0.1 on bivariate analysis in the multivariate model. We conducted statistical analysis in Stata version 16.0 (StataCorp LLC, https://www.stata.com) and R version 4.1.1 (The R Project for Statistical Computing, https://www.r-project.org) (*28*,*29*).

### Ethics Approval

The International Research Force Pakistan institutional review board reviewed and approved the survey protocol. This activity was reviewed by US Centers for Disease Control and Prevention and was conducted consistent with applicable federal law and Centers for Disease Control and Prevention policy (see e.g., 45 C.F.R. part 46.102(l)(2), 21 C.F.R. part 56; 42 U.S.C. §241(d); 5 U.S.C. §552a; 44 U.S.C. §3501 et seq.). All participants provided verbal consent. Participants were able to end the survey at any time for any reason.

## Results

We conducted phone interviews with 4,863 persons (response rate 20%, 4,863/24,315) and face-to-face interviews with 1,715 persons (response rate 70%, 1,715/,2450), for a total of 6,578 interviews. The median age of participants was 32 years. After weighting the data, we split the population roughly equally, 50.6% women and 49.4% men (Table 1). Geographically, 35.4% lived in rural areas and 64.6% lived in urban areas. Approximately half (49.9%) completed less than primary education, 19.8% primary and middle, 23.2% secondary and high, and 7.1% graduate and postgraduate. Approximately half (50.3%) were employed, 40.7% were female homemakers, and 9.1% were not working. In terms of income (thresholds were originally set in Pakistan rupees and converted to US dollars [USD]), 35.8% did not earn any money, 49.9% earned <$163 USD per month, 8.3% earned $163–$208 USD per month, and 5.9% earned >$208 USD monthly. The percentage who earned money is notably higher than those who were employed, which might be because some persons work in the informal sector and do not consider themselves to be employed.

**Table 1:** Characteristics of 6,577 COVID-19 survey participants in Pakistan, 2021–2022*

Characteristic	All, no. (%)	Categories, no. (%, 95% CI)			
		COVID-19 is inevitable, n = 1,828	COVID-19 is not inevitable, n = 3,980	Tested for COVID-19, n = 1,424	Have not tested for COVID-19, n = 5,113
Sex					
M	4,470 (49.4)	1,351 (32.0, 29.5–34.5)	2,504 (68.0, 65.5–70.5)	1,127 (22.1, 20.1–24.3)	3,311 (77.9, 75.7–79.9)
F	2107 (50.6)	477 (21.2, 19–23.5)	1,476 (78.8, 76.5–81)	297 (11.7, 10.1–13.5)	1,802 (88.3, 86.5–89.9)
Age, y					
18–24	1,415 (19)	409 (28.1, 24.5–31.9)	860 (71.9, 68.1–75.5)	249 (16.8, 13.9–20.2)	1,156 (83.2, 79.9–86.2)
25–34	2,239 (33.3)	587 (25.0, 22.3–28)	1,416 (75.0, 72–77.7)	529 (17.7, 15.4–20.1)	1,702 (82.3, 79.9–84.6)
35–44	1,553 (26.3)	428 (25.4, 22.2–29)	941 (74.6, 71.1–77.8)	343 (15.6, 13.2–18.4)	1,199 (84.4, 81.7–86.8)
>45	1,370 (21.4)	404 (28.0, 24.3–31.9)	763 (72.1, 68.1–75.7)	303 (17.1, 14.3–20.3)	1,056 (82.9, 79.7–85.7)
Residence					
Rural	3,715 (64.6)	641 (23.5, 21.3–25.9)	1,898 (76.5, 74.1–78.7)	907 (15.7, 14.0–17.7)	2,325 (84.3, 82.3–86.0)
Urban	2,862 (35.4)	1,187 (31.4, 29.1–33.8)	2,082 (68.6, 66.2–70.9)	517 (18.8, 17.1–20.7)	2,788 (81.2, 79.4–82.9)
Employment					
Employed	3,952 (50.3)	1,186 (29.5, 27.0–32.2)	2,214 (70.5, 67.8–73.0)	998 (21.1, 19.0–23.4)	2,927 (78.9, 76.6–81.0)
Not working	831 (9.1)	255 (29.9, 25.9–34.2)	495 (70.1, 65.8–74.1)	194 (21.1, 17.7–25.1)	630 (78.9, 74.9–82.4)
Female homemaker	1,673 (40.7)	362 (21.8, 19.5–24.4)	1,193 (78.2, 75.6–80.5)	202 (10.6, 8.9–12.5)	1,465 (89.4, 87.5–91.1)
Monthly income, USD					
None	1,429 (35.8)	334 (22.4, 19.6–25.4)	991 (77.7, 74.6–80.4)	222 (12.4, 10.4–14.7)	1,200 (87.6, 85.3–89.6)
<$163	2,668 (49.9)	823 (28.0, 25.4–30.8)	1,550 (72.0, 69.2–74.6)	527 (17.6, 15.5–20.0)	2,133 (82.4, 80.0–84.5)
$163–208	562(8.3)	196 (32.9, 27.2–39.3)	318 (67.1, 60.8–72.9)	144 (19.0, 14.7–24.3)	416 (81.0, 75.7–85.3)
>$208	494 (5.9)	154 (33.4, 26.5–41.0)	279 (66.6, 59.0–73.5)	165 (25.7, 20.6–31.6)	324 (74.3, 68.4–79.4)
Highest level of education completed					
Less than primary	1,731 (49.9)	413 (23.4, 20.7–26.3)	1,085 (76.6, 73.7–79.3)	267 (13.9, 11.9–16.3)	1,448 (86.1, 83.7–88.1)
Primary and middle	1,256 (19.8)	315 (26.2, 22.9–29.7)	799 (73.8, 70.3–77.1)	207 (14.8, 12.3–17.6)	1,048 (85.2, 82.4–87.7)
Secondary and high	2,316 (23.2)	661 (29.5, 26.9–32.2)	1,388 (70.5, 67.8–73.1)	546 (21.6, 19.4–24.0)	1,758 (78.4, 76.0–80.6)
Graduate and postgraduate	1,222 (7.1)	429 (36.3, 32.4–40.5)	677 (63.7, 59.5–67.6)	391 (27.2, 23.5–31.2)	820 (72.8, 68.8–76.5)
PHQ-4 mental health status†					
Normal	2,687 (45.1)	786 (27.7, 25.1–30.4)	1,662 (72.3, 69.6–74.9)	606 (18.6, 16.4–20.9)	2,076 (81.4, 79.1–83.6)
Mild	2,306 (36.9)	622 (24.2, 21.5–27.0)	1,362 (75.8, 73.0–78.5)	461 (13.7, 11.8–15.8)	1,822 (86.3, 84.2–88.2)
Moderate	685 (12.7)	234 (31.2, 26.2–36.8)	383 (68.8, 63.2–73.8)	145 (18.4, 14.4–23.2)	536 (81.6, 76.8–85.6)
Severe	332 (5.3)	82 (26.8, 19.8–35.2)	217 (73.2, 64.8–80.2)	64 (13.0, 9.1–18.3)	264 (87.0, 81.7–90.0)
Worried about COVID-19					
Very worried	1,477 (22.2)	534 (35.8, 31.8–39.9)	746 (64.2, 60.1–68.2)	342 (18.9, 16.0–22.1)	1,124 (81.1, 77.9–84.0)
Moderately	260 (3.7)	75 (29.8, 22.3–38.7)	144 (70.2, 61.4–77.7)	59 (15.4, 10.8–21.7)	200 (84.6, 78.3–89.2)
A little	1,540 (24.3)	472 (27.7, 24.6–31.0)	911 (72.3, 69.0–75.4)	275 (13.1, 11.1–15.4)	1,258 (86.9, 84.6–89.0)
Not at all	3,205 (49.9)	730 (21.2, 19.0–23.6)	2,131 (78.8, 76.4–81.0)	716 (17.5, 15.5–19.6)	2,473 (82.5, 80.4–84.5)
Tested for COVID-19					
Y	1,424 (16.8)	494 (36.6, 32.2–41.1)	772 (63.4, 58.9–67.8)	NA	NA
N	5,113 (83.2)	1,326 (24.2, 22.4–26.1)	3,188 (75.8, 74.0–77.6)	NA	NA
No. times tested					
1	138 (71.8)	NA	NA	NA	NA
2	35 (22.9)	NA	NA	NA	NA
3–5	10 (5.3)	NA	NA	NA	NA
Why tested					
Feel unwell	68 (39.4)	NA	NA	NA	NA
Contact with COVID-19 case	18 (12.7)	NA	NA	NA	NA
Required for work	39 (14.8)	NA	NA	NA	NA
Required for school	16 (7.9)	NA	NA	NA	NA
Other	32 (25.2)	NA	NA	NA	NA
COVID-19 inevitable					
Y	1,828 (26.3)	NA	NA	494 (23.6, 20.6–26.9)	1,326 (76.4,73.1–79.4)
N	3,980 (73.7)	NA	NA	772 (14.6, 13.1–16.3)	3,188 (85.4, 83.7–86.9)
Stressed due to additional responsibilities of caregiving or work due to COVID-19					
Strongly disagree	1,562 (24.2)	325 (19.5, 16.6–22.7)	1,068 (80.5, 77.3–83.4)	353 (17.3, 14.6–20.4)	1,193 (82.7, 79.6–85.4)
Disagree	854 (12.7)	279 (27.9, 23.4–32.8)	456 (72.2, 67.2–76.6)	148 (13.3, 10.3–17.1)	702 (86.7, 82.9–89.7)
Neutral	663 (10.9)	210 (31.7, 26.5–37.4)	378 (68.4, 62.6–73.6)	160 (17.7, 14.1–22.1)	501 (82.3, 77.9–85.9)
Agree	1,186 (20.6)	329 (23.6, 20.3–27.3)	748 (76.4, 72.7–79.7)	220 (11.9, 9.7–14.5)	962 (88.1, 85.5–90.3)
Strongly agree	2,157 (31.5)	651 (31.1, 27.9–34.4)	1,234 (68.9, 65.6–72.1)	503 (20.2, 17.7–23.0)	1,643 (79.8, 77.1–82.3)
COVID-19 is a serious health issue					
Y	4,915 (75.9)	1,514 (29.2, 27.3–31.2)	2,877 (70.8, 68.8–72.7)	1,101 (16.7, 15.2–18.2)	3,787 (83.4, 81.8–84.8)
N	1,340 (24.1)	251 (17.7, 14.6–21.3)	956 (82.3, 78.7–85.4)	252 (16.7, 13.7–20.3)	1,082 (83.3, 79.7–86.3)
Mask wearing					
Y	5,822 (85.7)	1,665 (27.3, 25.5–29.1)	3,493 (72.8, 70.9–74.5)	1,301 (17.4, 16.0–18.9)	4,486 (82.6, 81.1–84.0)
N	755 (14.3)	163 (20.5, 16.7–25.0)	487 (79.5, 75.0–83.3)	123 (13.6, 10.4–17.4)	627 (86.5, 82.6–89.6)
Physical distancing of 2 meters					
Y	3,904 (55.2)	1,212 (29.0, 26.7–31.3)	2,283 (71.0, 68.7–73.3)	849 (16.1, 14.4–17.9)	3,037 (83.9, 82.1–85.6)
N	2,673 (44.8)	616 (23.0, 20.6–25.6)	1,697 (77.0, 74.4–79.4)	575 (17.8, 15.8–20.0)	2,076 (82.2, 80.0–84.2)
Wash hands more frequently than before COVID-19					
Y	4,617 (67.5)	1,373 (28.0, 26.0–30.1)	2,790 (72.0, 69.9–74.0)	1,038 (17.2, 15.6–18.8)	3,555 (82.9, 81.2–84.4)
N	1,960 (32.5)	455 (22.6, 19.8–25.7)	1,190 (77.4, 74.3–80.2)	386 (16.2, 13.9–18.8)	1,558 (83.8, 81.2–86.1)
Avoid nonessential shopping					
Y	1,299 (22.3)	380 (26.4, 23.3–29.9)	840 (73.6, 70.1–76.7)	252 (13.2, 11.0–15.7)	1,047 (86.8, 84.3–89.0)
N	5,278 (77.8)	1,448 (26.3, 24.4–28.3)	3,140 (73.7, 71.7–75.6)	1,172 (17.9, 16.3–19.6)	4,066 (82.1, 80.5–83.7)
Avoid domestic travel					
Y	1,003 (19.0)	281 (26.5, 22.8–30.5)	660 (73.5, 69.5–77.2)	204 (12.4, 10.2–15.1)	799 (87.6, 84.9–89.8)
N	5,574 (81.0)	1,547 (26.3, 24.5–28.2)	3,320 (73.7, 71.8–75.6)	1,220 (17.9, 16.4–19.5)	4,314 (82.1, 80.5–83.6)
Avoid public transit					
Y	555 (11.7)	131 (21.8, 17.5–26.8)	400 (78.2, 73.2–82.5)	103 (11.7, 8.9–15.1)	451 (88.4, 84.9–91.1)
N	6,022 (88.3)	1,697 (27.0, 25.2–28.8)	3,580 (73.0, 71.2–74.8)	1,321 (17.5, 16.1–19.0)	4,662 (82.5, 81.0–83.9)
No change in precautions taken					
Y	306 (7.7)	59 (21.0, 14.0–30.1)	187 (79.0, 69.9–86.0)	53 (18.8, 12.3–27.7)	251 (81.2, 72.3–87.7)
N	4,557 (92.3)	1,444 (34.0, 31.6–36.6)	2,496 (66.0, 63.4–68.4)	1,188 (22.6, 20.7–24.7)	3,337 (77.4, 75.3–79.3)
Primary source of COVID-19 information					
Social media	2,591 (30.5)	766 (28.3, 25.5–31.2)	1,559 (71.8, 68.8–74.5)	634 (19.6, 17.4–22.0)	1,941 (80.4, 78.0–82.6)
Friends and family	984 (21.9)	221 (18.4, 15.4–21.8)	641 (81.6, 78.2–84.6)	156 (12.8, 10.2–16.0)	822 (87.2, 84.0–89.8)
TV	1,866 (36.6)	480 (26.0, 23.0–29.2)	1,144 (74.0, 70.9–77.0)	359 (14.5, 12.4–16.9)	1,500 (85.5, 83.1–87.6)
Other	770 (11.1)	284 (38.3, 32.9–44.0)	414 (61.7, 56.0–67.1)	207 (24.7, 20.2–29.8)	557 (75.3, 70.2–79.9)

*Numbers are unweighted, but percentages and corresponding confidence intervals are weighted. NA, not applicable. †Patient Health Questionnaire 4 scale: normal (0–2), mild (3–5), moderate (6–8), and severe (9–12). A score of higher than normal indicates anxiety and depression.

Mental health challenges were common; 36.9% of the weighted study population screened positive for mild anxiety and depression, 12.7% for moderate, and 5.3% for severe. Nearly half (49.9%) were not worried about COVID-19, 24.3% were a little worried, 3.7% were moderately worried, and 22.2% were very worried. There were 52.1% who noted that life was more stressful since the start of the pandemic because of additional caregiving or work responsibilities.

Most (75.9%) of the weighted study population thought COVID-19 was a serious health issue, and 26.3% thought contracting COVID-19 was inevitable. Men (32%) were more likely than women (21.2%) to think contracting COVID-19 was inevitable. Urban residents (31.4%) were more likely than rural residents (23.5%) to think contracting COVID-19 was inevitable. Of those who thought COVID-19 was inevitable, 36.6% had tested for COVID-19 and 24.2% had not. Of those respondents who thought COVID-19 was inevitable, almost one-third (29.2%) thought COVID-19 was serious compared with those who did not (17.7%). Among those respondents who thought COVID-19 was inevitable, 19.4% felt this made them less willing to avoid it, and 61.7% were more willing to try avoiding it (data not shown).

Fewer than 1 in 5 study participants (16.8%) had ever tested for COVID-19, with a higher proportion of men (22.1%) than women (11.7%) having tested. Testing was most common among those with the highest levels of income or education. One quarter (25.7%) of those earning >$208 USD per month had tested for COVID-19 compared with 12.4% of those who did not earn money. Similarly, 27.2% of those with a graduate or postgraduate degree had tested for COVID-19, compared with 13.9% of those with less than primary education. History of testing for COVID-19 was more common among those who thought getting COVID-19 was inevitable (23.6%) compared with those who thought it was not (14.6%). History of testing for COVID-19 also was more common for those with no anxiety or depression (18.6%) compared with those with severe anxiety or depression (13.0%). Of those who tested for COVID-19, 39.4% did so because they felt unwell, 14.8% because it was required for work, 12.7% because they were in close contact with someone with COVID-19, and 7.9% because it was required for school. Among those who tested for COVID-19, 71.8% tested once, 22.9% tested twice, and 5.3% tested 3–5 times.

In multivariate analysis, thinking COVID-19 was inevitable was associated with thinking it was a serious health issue (adjusted odds ratio [aOR] 1.7, 95% CI 1.2—.4) (Table 2). People living in rural areas were less likely to think COVID-19 was inevitable than urban residents (aOR 0.7, 95% CI 0.6—0.8). Thinking COVID-19 was inevitable was not associated with mask wearing, physical distancing, handwashing, or avoiding nonessential shopping, domestic travel, or public transport.

**Table 2:** Correlates of belief that COVID-19 infection is inevitable and having been tested for COVID-19, Pakistan, 2021–2022*

Characteristic	COVID-19 is inevitable†					Tested for COVID-19‡			
	OR (95% CI)	p value	aOR (95% CI)	p value		OR (95% CI)	p value	aOR (95% CI)	p value
Sex									
M	Referent	0	Referent	0.13		Referent	0	Referent	0.278
F	0.6 (0.5–0.7)		0.5 (0.3–0.9)			0.5 (0.4–0.6)		0.9 (0.6–1.4)	
Age, y									
18–24	Referent	0.909	NA	NA		Referent	0.797	NA	NA
25–34	0.9 (0.7–1.1)		NA			1.1 (0.8–1.4)		NA	
35–44	0.9 (0.7–1.1)		NA			0.9 (0.7–1.2)		NA	
≥45	1.0 (0.8–1.3)		NA			1.0 (0.8–1.4)		NA	
Residence									
Rural	0.7 (0.6–0.8)	0	0.7 (0.6–0.9)	0.001		0.8 (0.7–1.0)	0.021	0.8 (0.6–1.0)	0.078
Urban	Referent		Referent			Referent		Referent	
Employment									
Employed	Referent	0	Referent	0.102		Referent	0	Referent	0.100
Not working	1.0 (0.8–1.3)		0.9 (0.7–1.4)			1.0 (0.8–1.3)		1.0 (0.7–1.4)	
Female homemaker	0.7 (0.6–0.8)		2.4 (1.3–4.4)			0.4 (0.4–0.6)		0.6 (0.3–0.9)	
Monthly income, USD									
None	0.7 (0.6–0.9)	0.008	0.8 (0.5–1.2)	0.124		0.7 (0.5–0.9)	0.004	1.2 (0.8–1.7)	0.105
<$163	Referent		Referent			Referent		Referent	
$163–208	1.3 (0.9–1.7)		1.5 (1.0–2.2)			1.1 (0.8–1.6)		1.2 (0.8–1.7)	
>$208	1.3 (0.9–1.8)		1.0 (0.6–1.5)			1.6 (1.2–2.2)		1.5 (1.0–2.2)	
Highest level of education completed									
Less than primary	Referent	0	Referent	0.223		Referent	0	Referent	0.006
Primary & middle	1.2 (0.9–1.5)		1.2 (0.8–1.8)			1.1 (0.8–1.4)		1.0 (0.7–1.4)	
Secondary & high	1.4 (1.1–1.7)		1.1 (0.8–1.6)			1.7 (1.4–2.1)		1.3 (1.0–1.8)	
Graduate and postgraduate	1.9 (1.5–2.4)		1.2 (0.8–1.8)			2.3 (1.8–3.0)		1.5 (1.1–2.2)	
Patient Health Questionnaire 4 mental health status§									
Normal	Referent	0.843	NA	NA		Referent	0.082	Referent	0.048
Mild	0.8 (0.7–1.0)		NA			0.7 (0.6–0.9)		0.7 (0.5–0.9)	
Moderate	1.2 (0.9–1.6)		NA			1.0 (0.7–1.4)		0.9 (0.6–1.4)	
Severe	1.0 (0.6–1.4)		NA			0.7 (0.4–1.0)		0.5 (0.3–1.0)	
Worried about COVID-19									
Very worried	Referent	0	Referent	0.024		Referent	0.548	NA	NA
Moderately	0.8 (0.5–1.2)		0.9 (0.5–1.8)			0.8 (0.5–1.2)		NA	
A little	0.7 (0.5–0.9)		1.4 (0.9–2.0)			0.6 (0.5–0.9)		NA	
Not at all	0.5 (0.4–0.6)		0.7 (0.5–1.0)			0.9 (0.7–1.2)		NA	
Tested for COVID-19									
Y	1.8 (1.5–2.2)	0	1.2 (0.9–1.6)	0.381		NA	NA	NA	NA
N	Referent		Referent			NA		NA	
COVID-19 inevitable									
Y	NA	NA	NA	NA		1.8 (1.5–2.2)	0	1.5 (1.2–1.9)	0.001
N	NA		NA			Referent		Referent	
Stressed due to additional responsibilities of caregiving or work due to COVID-19									
Strongly disagree	Referent	0	Referent	0.061		Referent	0.083	Referent	0.803
Disagree	1.6 (1.2–2.2)		2.4 (1.5–3.7)			0.7 (0.5–1.0)		0.9 (0.6–1.4)	
Neutral	1.9 (1.4–2.6)		2.2 (1.4–3.5)			1.0 (0.7–1.4)		1.5 (1.0–2.2)	
Agree	1.3 (1.0–1.7)		2.3 (1.5–3.5)			0.6 (0.5–0.9)		0.8 (0.5–1.2)	
Strongly agree	1.9 (1.5–2.4)		1.8 (1.2–2.5)			1.2 (0.9–1.6)		1.1 (0.8–1.5)	
COVID-19 is a serious disease									
Y	1.9 (1.5–2.5)	0	1.7 (1.2–2.4)	0.001		1.0 (0.8–1.3)	0.960	NA	NA
N	Referent		Referent			Referent		NA	
Wearing a face mask									
Y	1.4 (1.1–1.9)	0.007	1.2 (0.7–1.9)	0.971		1.3 (1.0–1.8)	0.066	1.4 (0.9–2.1)	0.206
N	Referent		Referent			Referent		Referent	
Physical distance of 2 meters									
Y	1.4 (1.1–1.6)	0.001	1.3 (1.0–1.8)	0.103		0.9 (0.7–1.1)	0.222	NA	NA
N	Referent		Referent			Referent		NA	
Wash hands more frequently than before COVID-19									
Y	1.3 (1.1–1.6)	0.004	1.0 (0.7–1.5)	0.992		1.1 (0.9–1.3)	0.522	NA	NA
N	Referent		Referent			Referent		NA	
Avoid non-essential shopping									
Y	1.0 (0.8–1.2)	0.939	NA	NA		0.7 (0.6–0.9)	0.002	0.9 (0.7–1.2)	0.411
N	Referent		NA			Referent		Referent	
Avoid domestic travel									
Y	1.0 (0.8–1.3)	0.925	NA	NA		0.7 (0.5–0.8)	0.001	0.9 (0.6–1.2)	0.432
N	Referent		NA			Referent		Referent	
Avoid public transit									
Y	0.8 (0.6–1.0)	0.054	0.6 (0.3–1.1)	0.148		0.6 (0.5–0.9)	0.003	1.0 (0.7–1.5)	0.843
N	Referent		Referent			Referent		Referent	
No change in precautions taken									
Y	0.5 (0.3–0.8)	0.008	0.9 (0.4–1.9)	0.315		0.8 (0.5–1.3)	0.374	NA	NA
N	Referent		Referent			Referent		NA	
Primary source of COVID-19 information									
Social media	Referent	0.029	Referent	0.086		Referent	0.954	NA	NA
Friends and family	0.6 (0.4–0.7)		0.8 (0.5–1.3)			0.6 (0.4–0.8)		NA	
TV	0.9 (0.7–1.1)		1.1 (0.8–1.5)			0.7 (0.5–0.9)		NA	
Other	1.6 (1.2–2.1)		1.5 (1.0–2.1)			1.3 (1.0–1.8)		NA	

*aOR, adjusted odds ratio; NA, not applicable; OR, odds ratio.

†Variables included in the model for thinking COVID-19 is inevitable were: sex, residence, employment status, income, education, worried about contracting COVID-19, tested for COVID-19, stressed due to additional caregiving responsibilities, thinking COVID-19 is a serious disease, wearing a face mask, physical distancing, washing hands more frequently, avoid public transit, no change in precautions taken to prevent to COVID-19 transmission, primary source for COVID-19 information.

‡Variables included in the model for having tested for COVID-19 were: sex, residence, employment status, income, education, PHQ-4 status, thinking contracting COVID-19 is inevitable, stressed due to additional caregiving responsibilities, wearing a face mask, avoiding non-essential shopping, avoiding domestic travel and avoiding public transit.

§Patient Health Questionnaire-4 (PHQ-4) scale: normal (0–2), mild (3–5), moderate (6–8), and severe (9–12). A score of higher than normal indicates anxiety and depression.

From the testing of correlates of having tested for COVID-19, those with a graduate or postgraduate degree were more likely to have tested for COVID-19 (aOR 1.5, 95% CI 1.1—2.2) compared with those with less than primary education (Table 2). Female homemakers (aOR 0.6, 95% CI 0.3–0.9) were less likely to have tested than women who were employed. Testing for COVID-19 was not associated with mask wearing, physical distancing, handwashing, or avoiding nonessential shopping, domestic travel, or public transport.

We estimated the cumulative number of suspected and confirmed cases of COVID-19. Among household members of study participants, there were 316 confirmed cases, 856 suspected and confirmed cases, 24 caregiver deaths, and 2 children who were orphaned because of the virus (Table 3). Adjusting for Pakistan’s population, we estimated 1,518,000 (95% CI 880,000–2,156,000) confirmed cases and 4,180,000 (95% CI 3,256,000–5,192,000) suspected and confirmed cases.

**Table 3:** Survey-based estimates of confirmed and suspected COVID-19 cases, Pakistan, 2021–2022

COVID-19 cases	No.	Illness rate (95% CI)	No. cases/100,000 population	Total estimated no. cases (95% CI)
Confirmed cases	316	0.0069 (0.0040–0.0098)	690	1,518,000 (880,000–2,156,000)
Suspected and confirmed cases	856	0.0192 (0.0148–0.0236)	1,900	4,180,000 (3,256,000–5,192,000)

## Discussion

Our nationally representative COVID-19 survey in Pakistan explores views on the inevitability of contracting COVID-19, gauges public tendency to seek out testing, and estimates the number of COVID-19 cases. Compared with those living in rural areas, urban residents were more likely to think COVID-19 was inevitable. Completion of a graduate degree, being employed, and screening positive for anxiety and depression were associated with having tested for COVID-19. Our estimates of the number of confirmed cases were 17.7% higher than official estimates of confirmed cases: 1,518,000 compared with 1,290,000. Our estimates of suspected cases were nearly 3 times as high as official estimates: 4,180,000 compared with 1,290,000 (*6*).

Although the initial spread of COVID-19 in Pakistan was first recognized in urban areas, incidence in rural areas was equal to that in urban areas (*30*). Nonetheless, we found that rural residents were less likely than urban residents to consider COVID-19 inevitable. It is possible that residents in rural areas are more likely to live in less densely populated settings or work outside, scenarios where physical distancing is more easily accomplished and transmission is less likely (*31*). Feeling that COVID-19 is inevitable was not associated with practicing prevention behaviors (e.g., indoor mask wearing, maintaining physical distancing, handwashing, avoiding nonessential shopping, domestic travel, and taking public transportation) suggests there are opportunities to promote and support prevention behaviors even among those resigned to getting COVID-19.

Attitudes about the seriousness of COVID-19 as a health issue were related to attitudes about its inevitability; those who thought it was a serious health issue were more likely to think they would inevitably contract COVID-19. An April 2021 convenience survey in Peshawar, Pakistan, found that 66% of persons thought COVID-19 was a serious health issue (*32*). We found 75.9% of persons thought it was a serious health issue. As more persons become infected with SARS-CoV-2 and more know someone who became seriously ill or died, it is possible that more persons may also think the disease is serious. This situation could change, however, with the arrival of new variants, such as Omicron, that may result in less severe illness (*33*). The Delta variant, which was more transmissible than previous variants but induced the same level of disease severity, arrived in Pakistan after the mobile phone survey but before completion of the face-to-face survey (*34*).

Testing is a critical tool for both COVID-19 surveillance and mitigation. More than 1 year into the pandemic, less than one fifth of Pakistan’s population (16.8%) had been tested for COVID-19. Education level and employment status were significantly associated with having tested for COVID-19; those with graduate or postgraduate degrees and those who were employed were more likely to have tested than those with less than a primary education or who were not working. Although ≈70% of Pakistan residents access health care at private health facilities, both education and employment status were associated with accessing health care at private health facilities in Pakistan (*35*). As of March 7, 2022, a total of 82% of the 239 COVID-19 testing sites in Pakistan were in private or mixed public-private health facilities (*36*). COVID-19 testing is free of charge at public health facilities, but there is a cost to test at private health facilities. Assuming therefore that residents with higher socioeconomic status would be more likely to have tested for COVID-19, reported testing results might provide an incomplete picture of COVID-19 incidence and, consequently, deaths, and are likely not reflective of the entire population.

Increasing testing likely requires increasing both supply and demand in Pakistan. Although three quarters felt that COVID-19 was a serious health issue, a national survey conducted in March 2021 found that 28% of persons surveyed would do nothing if they had COVID-19 symptoms, 27% would isolate at home, 18% would treat themselves, 14% would get tested, and 6% would go to a clinic (*37*). Those data suggest that although people consider COVID-19 a serious health issue, they may think it is a serious health issue for others and not for themselves, making them inclined to avoid confirming their illness and not seek necessary treatment. Determining whether people understand their own risks for severe illness from COVID-19 and why they avoid getting tested for COVID-19 would help to inform COVID-19 policy making. Possible reasons could be that testing facilities are far or busy. One of the main barriers to accessing public healthcare services in Pakistan is the long wait times, as well as cost (*38*).

Overall, we found that COVID-19 prevention behaviors of mask wearing, physical distancing, handwashing, and avoiding nonessential shopping, domestic travel and public transit were not associated with thinking COVID-19 is inevitable or getting tested for COVID-19. Messaging about how these behaviors can help to protect family and friends might help to encourage people to engage in them.

Our estimates of suspected and confirmed cases are nearly 3 times higher than the number of officially confirmed cases, highlighting the low availability, access, and uptake of COVID-19 testing. Such a disparity in regard to the incidence of COVID-19 in Pakistan also suggests a more substantial loss of caregivers and indicates that the impact of the COVID-19 pandemic on families might be underestimated.

Responses to our survey were self-reported, so there is some risk for inaccurate responses because of recall bias or other reasons. Some participants completed the survey using a mobile phone and others provided responses in face-to-face interviews, which could also bias responses. Because of funding delays, there was a gap of approximately 4 months between the end of the mobile phone survey and beginning of the face-to-face survey. Incidence was much higher during the mobile phone survey than the face-to-face survey, which might have influenced responses because persons might be more likely to engage in mitigation measures when cases are high. Conversely, the face-to-face survey happened later, when pandemic fatigue might have begun to emerge across the population, possibly leading residents to relax mitigation behaviors. Emergence of the Delta variant during that time could also have influenced responses.

We determined that most people in Pakistan engage in prevention behaviors and consider COVID-19 a serious health issue. Unfortunately, our survey of Pakistan residents also demonstrated that there is substantial undertesting and thus underreporting of COVID-19 incidence and deaths. Further research is needed to understand why so few persons are getting tested and to determine whether they truly understand the risk of COVID-19 to themselves and to those around them.
